# Bruton’s Tyrosine Kinase: An Emerging Key Player in Innate Immunity

**DOI:** 10.3389/fimmu.2017.01454

**Published:** 2017-11-08

**Authors:** Alexander N. R. Weber, Zsofia Bittner, Xiao Liu, Truong-Minh Dang, Markus Philipp Radsak, Cornelia Brunner

**Affiliations:** ^1^Department of Immunology, Interfaculty Institute for Cell Biology, University of Tübingen, Tübingen, Germany; ^2^Department of Internal Medicine III, University Medical Center of the Johannes Gutenberg-University Mainz, Mainz, Germany; ^3^Department of Otorhinolaryngology, Ulm University Medical Center, Ulm, Germany

**Keywords:** Bruton’s tyrosine kinase, macrophage, dendritic cell, Toll-like receptor, NLRP3 inflammasome, ibrutinib, X-linked agammaglobulinemia

## Abstract

Bruton’s tyrosine kinase (BTK) was initially discovered as a critical mediator of B cell receptor signaling in the development and functioning of adaptive immunity. Growing evidence also suggests multiple roles for BTK in mononuclear cells of the innate immune system, especially in dendritic cells and macrophages. For example, BTK has been shown to function in Toll-like receptor-mediated recognition of infectious agents, cellular maturation and recruitment processes, and Fc receptor signaling. Most recently, BTK was additionally identified as a direct regulator of a key innate inflammatory machinery, the NLRP3 inflammasome. BTK has thus attracted interest not only for gaining a more thorough basic understanding of the human innate immune system but also as a target to therapeutically modulate innate immunity. We here review the latest developments on the role of BTK in mononuclear innate immune cells in mouse versus man, with specific emphasis on the sensing of infectious agents and the induction of inflammation. Therapeutic implications for modulating innate immunity and critical open questions are also discussed.

## Introduction

**Figure 1:**
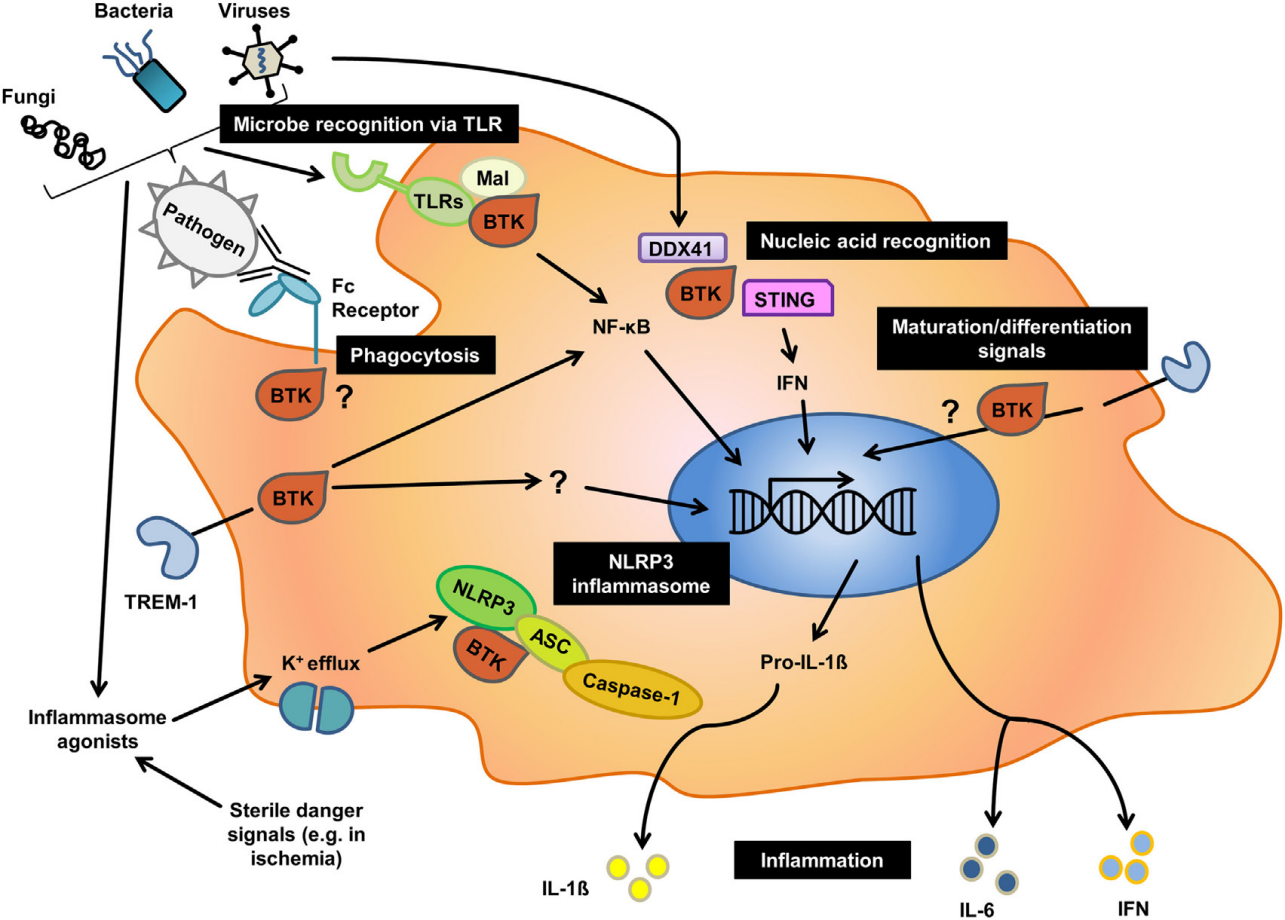
Overview of the different roles of Bruton’s tyrosine kinase (BTK) in innate immunity. Black boxes indicate major cellular processes for which an involvement of BTK has been reported in human or mice, or both. As outlined in the text, for processes such as phagocytosis there is contradictory evidence illustrating that the nature of function-modifying mutations, cellular context and species may have a profound effect on the role of BTK in a given process.

Since the first description of X-linked agammaglobulinemia (XLA, OMIM entry 300300) (1) and the identification of Bruton’s tyrosine kinase (*BTK*) as its genetic cause (2), BTK has been widely characterized as a critical mediator of B cell receptor (BCR) signaling and thus adaptive immunity (3). In the murine *Btk-*mutated (R28C) X-linked immunodeficiency (*Xid)* mutant strain CBA/N (4) B cell numbers and functionality are reduced but detectable [e.g., unaffected B-1b cell levels (5)]. In contrast, in humans BTK’s pivotal role is highlighted by the fact that a wide spectrum of *BTK* loss-of-function mutations [reviewed by Ref. (6) and documented in the ‘BTKbase’ database] lead to an almost complete absence of peripheral B cells and antibodies in XLA. BTK catalytic activity typically drives the activation of at least three key signaling pathways, phospholipase C, phosphatidalyinositol-3-kinase/Akt and NF-κB, giving B cells a very strong survival signal upon BCR engagement. Totaling a molecular weight of approximately 77 kDa, BTK also contains an N-terminal Pleckstrin homology domain that binds membrane phosphatidylinositol (3,4,5)-trisphosphate (PIP_3_), and Tec homology, Src homology (SH) 3, and SH2 domains involved in protein-protein interactions. Y223 and Y551 represent two critical tyrosine phosphorylation sites in the SH3 and kinase domain (7). Y551 is phosphorylated by the kinases Syk or Lyn during BCR signaling and promotes the catalytic activity of BTK and subsequent Y223 autophosphorylation. The strong dependence of malignant B cells on BTK activity for survival (3), made BTK a key target for the development of small molecule inhibitors (8) in B cell malignancies. Nevertheless, BTK is being increasingly studied for its role in myeloid and other innate immune cells (Figure 1). Here, we summarize the emerging multi-faceted roles of this versatile and therapeutically tractable kinase in innate immunity.

## BTK in Infection and Danger Recognition by Cell Surface Receptors in Innate Immune Cells

Although innate immune contributions for BTK in *in vivo* infection models with *Btk* gene knockout or *Xid* mice have to be interpreted with care (see below), a role for BTK/Btk in the sensing of multiple microbes has been reported: Sensing and antimicrobial responses to *Listeria monocytogenes* (9), *Staphylococcus aureus* (10), dengue virus (11), and *Aspergillus fumigatus* (12) were shown to depend on BTK. This effect may in part be due to BTK’s involvement in the sensing of microbes *via* multiple Toll-like receptors (TLRs)—TLR2 (13, 14), TLR3 (11), TLR4 (14, 15), TLR7/8 (14, 16, 17), and TLR9 (9, 17, 18) on human and mouse macrophages and dendritic cells (DC). However, some TLR studies, especially those involving XLA patients, have been contradictory with regard to specific TLRs requiring BTK (19). Potentially, the functional requirements for BTK function during B cell development are higher, leading to an XLA phenotype in a broader range of mutations and thus patients; conversely, it seems that for TLR signaling only certain BTK mutations may cause a significant impairment of signaling. Within the vast spectrum of BTK mutations reported in XLA patients the functional impact can oftentimes not adequately be predicted. On a postreceptor level, BTK is thought to interface with canonical TLR pathways at the level of the TLR/MyD88 bridging adaptor Mal/TIRAP, one suggested direct BTK substrate (15, 20, 21) apart from TLR3 (11). TLR-dependent BTK-activation promotes NF-κB and interferon-regulatory factor-dependent transcription of inflammatory cytokines and interferons (IFNs) (15, 17). BTK was also linked with the cytosolic nucleic acid sensor DDX41 (11) and promoted its cooperation with the important IFN response regulator STING. BTK also operates downstream of the myeloid receptor TREM-1 for cytokine production (22, 23). On a more global immunoregulatory level, downregulation of innate immune-related genes and an upregulation of oxidative phosphorylation and apoptosis-related genes was observed in XLA patients (24). In contrast to these proimmune innate functions of BTK, the kinase was also shown to negatively regulate TLR-induced cytokine release from primary human innate immune cells (25). Moreover, in other DC studies, hepatocyte growth factor (HGF) as well as T cell Ig and mucin protein-3 (TIM-3)-induced BTK function blocked NF-κB activity (26, 27). In phagocytosis BTK was found essential for the clearance of infectious agents by mouse macrophages (12, 28); for humans, both data supporting a requirement for BTK in phagocytosis (24, 29, 30) as well as data arguing for a redundant role of BTK in this process (19, 31) have been reported based on studies of cells from XLA patients. Off-target effects in studies involving BTK inhibitors and the aforementioned unpredictability of naturally occurring BTK mutations or gene alterations^1^ are likely to contribute to these controversial findings. The breadth of this multifaceted body of evidence certainly highlights the complexity of BTK function and regulation. Specific mutation site, receptor pathway, cell type and species are thus important factors, rendering the more systematic exploration of BTK’s role in innate immunity a formidable challenge.

## BTK in the Maturation, Recruitment and Function of Innate Immune Cells

Given its role in B cell development, a role for BTK in the development of myeloid cells, which depends on many cues provided by cell surface receptors (32), is not surprising. Interestingly, in mice GM-CSF receptor α-chain expression was required for macrophage maturation and survival. In mice, Btk deficiency also correlated with reduced monocyte/macrophage numbers (33) but favored granulopoiesis (34, 35). However, these granulocytes were immature, had inefficient granule function and impaired recruitment of neutrophils to sites of sterile inflammation. Similarly, in humans BTK seems to be implicated in the maturation of neutrophils, since in XLA patients, who are frequently neutropenic, neutrophils were arrested at the myelocyte/promyelocyte stage (36–38). Conversely, Marron et al. (19) and Cavaliere et al. (31) suggested that BTK is dispensable for human neutrophil function; Honda et al. (39) even found an increased TLR or tumor necrosis factor receptor-induced ROS production of XLA neutrophils, albeit at higher levels of neutrophil apoptosis. Although DC numbers in *Btk*-deficient animals were unaffected, these DC had defects in maturation and DC-mediated antigen presentation (40). In human DC, the aforementioned HGF- and TIM-3-induced BTK-mediated NF-κB inhibition impaired DC activation as well as maturation leading to impaired CpG-induced anti-tumor responses (26, 27). In tumor infiltrating macrophages BTK was found to exert immune-inhibitory and tumor-promoting effects (41, 42). In contrast, inhibition of Btk activity promoted DC maturation and CD4^+^ T cell activating functions (43, 44). Together, these data suggest BTK may serve as an important target for immunomodulatory-based anticancer therapy. The unexpected description of (so far) cancer-specific alternative isoforms, p65 and p80, in breast (45), brain (46), prostate (47), gastric (48), and colon cancer (49) as well as reports for a role of BTK in NK cells (50), and platelets (51) also deserve mention and warrant further research.

## BTK and the NLRP3 Inflammasome

**Figure 2:**
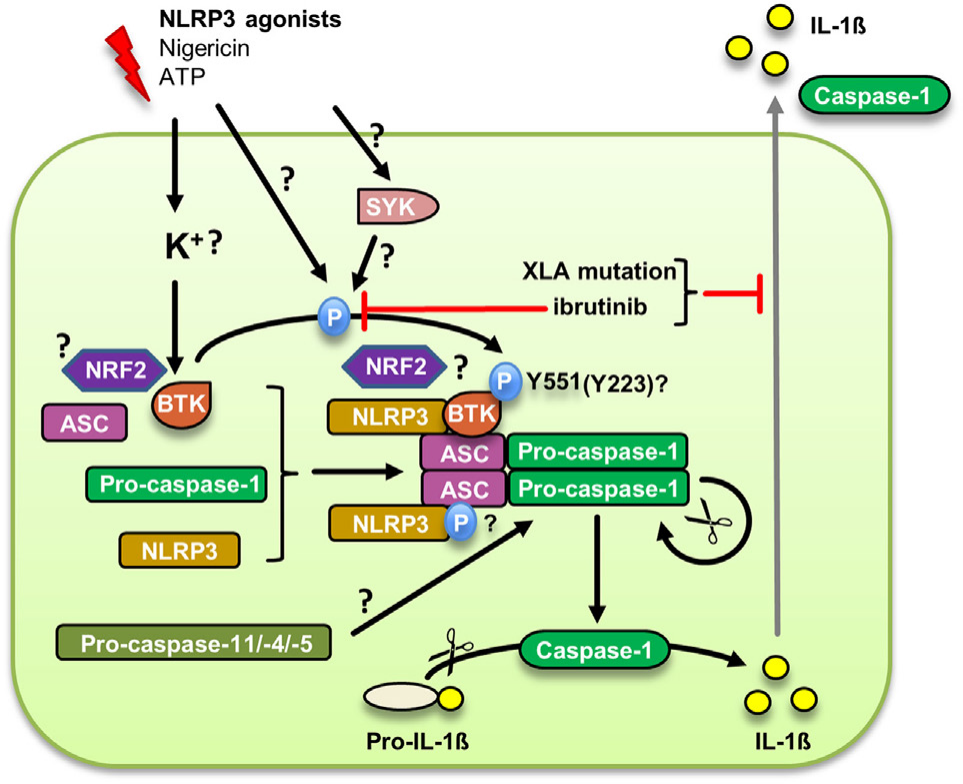
Bruton’s tyrosine kinase (BTK) regulation of the canonical NLRP3 inflammasome. Upon an upstream signal potentially linked to membrane integrity or K^+^ efflux, BTK is phosphorylated at Y551, presumably by Syk, and subsequently is activated. The supposed phosphorylation of ASC promotes inflammasome assembly and caspase-1 autoproteolytic activation leading to the cleavage and secretion of mature IL-1β. Whether BTK also plays a role in the alternative NLRP3 inflammasome dependent on caspase-11 remains to be investigated.

The NLRP3 inflammasome, a multiprotein complex involving NLRP3, the adaptor ASC and the proteolytic enzyme, caspase-1, has recently emerged as a key molecular machinery for the processing and thus activation of bioactive IL-1β (52, 53) and a major pathophysiological regulator in infection, myocardial infarction, stroke, Alzheimer’s and diabetes (53). Reports by us (10) and others (54) recently identified BTK as a direct regulator in NLRP3 inflammasome activation (Figure 2): Ito et al. demonstrated that BTK was critically required for NLRP3 inflammasome-dependent IL-1β release from murine macrophages. BTK physically interacted with NLRP3 and its adaptor ASC, resulting in the induction of ASC oligomerization and caspase-1 activation in a kinase activity-dependent manner *in vitro*. In both studies, BTK was rapidly phosphorylated upon NLRP3 activation. We additionally observed that inflammasome activity was impaired in PBMC from XLA patients, suggesting that a genetic inflammasome deficiency may contribute to the immunocompromised XLA phenotype. Pharmacological BTK inhibitors *in vivo* affected *S. aureus* clearance in mice and IL-1β release in cancer patients, which was associated with a reduced ability of isolated PBMC to secrete IL-1β. Excessive IL-1β release in PBMC from Muckle-Wells Syndrome MWS (OMIM entry 191900) patients could also be blocked by BTK inhibitors (10). In a brain ischemia/reperfusion *in vivo* model Btk was activated in infiltrating macrophages/neutrophils, and Btk inhibition protected against brain injury (54). In combination, these results warrant the exploration of BTK inhibition as a strategy to target the NLRP3 inflammasome therapeutically. Mechanistically, the emerging role of NRF2, a protein shown separately to interact with both BTK (55) and NLRP3 (56), will also be interesting to study further. Likewise, the observed link with caspase-11 (33) may indicate an additional role for BTK in the non-canonical NLRP3 inflammasome that depends on caspase-11 in mice and caspase-4/-5 in humans for intracellular LPS sensing (57)—a notion intriguing for further study.

## Therapeutic Opportunities in Innate Immunity

Undoubtedly, the existence of and first clinical data for an FDA-approved BTK inhibitor, ibrutinib (also known as PCI-32765), in oncology (8) make preclinical and translational research into BTK’s innate functions highly interesting, for example in arthritis (30), thromboinflammation (51), or in ischemic stroke, as aforementioned (52, 54). Compared to other strategies proposed to target the pathologically relevant NLRP3 inflammasome/IL-1 axis—for example, the inhibitor MCC950, whose target is however unknown (58), or IL-1 blockade which only neutralizes the inflammatory potential of certain inflammasome-dependent mediators—targeting NLRP3 *via* BTK is highly intriguing since BTK is a well-known (if incompletely understood) molecular target with inhibitors approved or in clinical trials. In cancer immunotherapies, first results on BTK inhibition modulating DC and subsequent CD4^+^ T cell activation (43) or upregulation of the inhibitory receptor TIM-3 on DCs are also noteworthy (59). On the other hand, targeting BTK with ibrutinib causes significant immunosuppression associated with an increased risk of infections (60) indicating that BTK dependent innate immunity is severely impaired (23). In addition, leukostasis as well as bleeding complications have been reported indicating that BTK inhibition by ibrutinib also affects leukocyte adhesion and platelet functions in a clinically relevant way (51, 61). Increased rates of atrial fibrillation (62) as a non-immune adverse event in patients receiving ibrutinib advises caution when exploring the novel opportunities of BTK blockade in various disease entities. Potentially, transient use of inhibitors, e.g., only during phases of acute adverse inflammation (e.g., shortly after ischemic brain or heart injury), may nevertheless offer advantageous therapeutic windows in non-chronic diseases. Nonetheless, much further work will be required to safely harness the potential of BTK for treating additional innate immune-related disorders.

## Open Questions and Outlook

Although much progress on deciphering the molecular function of BTK in various innate cell types has been made, specific BTK interactors and substrates in the different aforementioned processes have to be studied more systematically as highlighted by the many apparent controversies. Additionally, whether BTK functions as a *bona fide* kinase or more as a scaffold protein requires clarification, e.g., in the NLRP3 inflammasome process. In cell lines, well-characterized loss and gain of function mutants of BTK may be useful tools (22). Conditional and/or inducible gain- or loss-of-function mouse alleles, which surprisingly have not been described, will be essential for innate immunologists to meaningfully study BTK further *in vivo* and to exclude confounding effects from impaired B cell function, e.g., in *in vivo* infection studies. Furthermore, conditional alleles would help flesh out cell-specific and hematopoietic roles of BTK more precisely. The resulting *in vivo* mouse models should complement urgently needed additional studies on human BTK that may help to solve some of the apparent discrepancies between human and murine studies and decipher some of the profound complexity surrounding BTK. Such vital research could be done within ongoing studies in the cancer field or of *ex vivo* studies on biomaterial from healthy volunteers or XLA patients. Concomitant and standardized kinase and expression level assays conducted on XLA samples may help to gauge the penetrance and severity of naturally occurring variants better and, by incorporating these results, may allow drawing more generally valid conclusions from these patient studies.

In conclusion, BTK has emerged as a key node in many immunological signaling networks in innate immunity, some of which have profound therapeutic potential. Future efforts in both academia and industry may help to explore and subsequently harness the potential of this intriguing yet highly complex kinase for innate immunity. This may offer therapeutic opportunities comparable or potentially exceeding those already envisaged for oncology.

## Author Contributions

All authors collected and analyzed data, AW coordinated the study and drafted the manuscript, and all authors contributed toward and approved the final manuscript.

## Conflict of Interest Statement

The authors declare that the research was conducted in the absence of any commercial or financial relationships that could be construed as a potential conflict of interest.

## Footnotes

^1^Gross deletion within the *BTK* genomic locus could affect not only expression and function of BTK itself but also that of adjacent genes like *TIMM8A*, the genetic cause of the Mohr-Tranebjærg syndrome (MTS, OMIM entry 304700), a neurodegenerative disorder leading to sensorineural deafness. Additionally, beside isolated *BTK*-deficiency (XLA OMIM entry 300300), patients were reported with growth hormone deficiency (GHD) associated with mutations within the BTK gene (XLH-GHD, OMIM entry 307200). The reason for GHD in XLA remains obscure.
